# Validation of Clinical Risk Models for Clostridioides difficile*-*Attributable Outcomes

**DOI:** 10.1128/aac.00676-22

**Published:** 2022-06-21

**Authors:** Gregory R. Madden, William A. Petri, Deiziane V. S. Costa, Cirle A. Warren, Jennie Z. Ma, Costi D. Sifri

**Affiliations:** a Division of Infectious Diseases & International Health, Department of Medicine, University of Virginia grid.27755.32grid.412587.d School of Medicine, Charlottesville, Virginia, USA; b Department of Public Health Sciences, University of Virginia grid.27755.32grid.412587.d School of Medicine, Charlottesville, Virginia, USA; c Office of Hospital Epidemiology/Infection Prevention & Control, UVA Health, Charlottesville, Virginia, USA

**Keywords:** outcome, disease severity, *Clostridioides difficile*, disease severity, prediction model, risk model

## Abstract

Clostridioides difficile is the leading health care-associated pathogen, leading to substantial morbidity and mortality; however, there is no widely accepted model to predict C. difficile infection severity. Most currently available models perform poorly or were calibrated to predict outcomes that are not clinically relevant. We sought to validate six of the leading risk models (Age Treatment Leukocyte Albumin Serum Creatinine (ATLAS), C. difficile Disease (CDD), Zar, Hensgens, Shivashankar, and C. difficile Severity Score (CDSS)), guideline severity criteria, and PCR cycle threshold for predicting C. difficile-attributable severe outcomes (inpatient mortality, colectomy/ileostomy, or intensive care due to sepsis). Models were calculated using electronic data available within ±48 h of diagnosis (unavailable laboratory measurements assigned zero points), calibrated using a large retrospective cohort of 3,327 inpatient infections spanning 10 years, and compared using receiver operating characteristic (ROC) and precision-recall curves. ATLAS achieved the highest area under the ROC curve (AuROC) of 0.781, significantly better than the next best performing model (Zar 0.745; 95% confidence interval of AuROC difference 0.0094–0.6222; *P = *0.008), and highest area under the precision-recall curve of 0.232. Current IDSA/SHEA severity criteria demonstrated moderate performance (AuROC 0.738) and PCR cycle threshold performed the worst (0.531). The overall predictive value for all models was low, with a maximum positive predictive value of 37.9% (ATLAS cutoff ≥9). No clinical model performed well on external validation, but ATLAS did outperform other models for predicting clinically relevant C. difficile*-*attributable outcomes at diagnosis. Novel markers should be pursued to augment or replace underperforming clinical-only models.

## INTRODUCTION

Clostridioides difficile infection (CDI) continues to be an often morbid or lethal condition. Despite available treatments, up to a third of hospitalized cases will require intensive care, 3% a colectomy, and 6% will die (1). Evidence suggests that earlier interventions such as colectomy (2) and promising new treatments (e.g., fecal microbiota transplant, monoclonal antibodies [3], SER-109 [4], antisense antibiotics [5]) may prevent severe outcomes and death in selected patients; however, clinicians and researchers lack a valid and reliable method to risk stratify patients for these interventions early, at the time of diagnosis. Conversely, identifying patients at very low risk for serious adverse outcomes could help the significant issue of C. difficile overdiagnosis and overtreatment (6, 7).

A variety of published outcome models (Table 1) have been derived within small populations (e.g., Belmares et al., 102 patients at 1 hospital) (8), utilize trial cohorts with exclusion criteria that limit generalizability (e.g., ATLAS) (9), use variables occurring *after* severe outcomes have begun to develop (e.g., Im et al., imaging findings up to a week after diagnosis) (10), and/or were calibrated to predict outcome measures that are nonspecific to CDI (e.g., Kulaylat et al., all-cause mortality (1)). The few models that are validated at multiple institutions do not perform well on external validation (11–14). In addition, simplified clinical factors or laboratory cutoffs based on expert opinion including those recommended by the current Infectious Disease Society of America and Society for Healthcare Epidemiology of America (IDSA/SHEA) consensus guidelines (15, 16) do not perform well either (17), especially in patients with disrupted immunity (e.g., neutropenia) or kidney disease (18). As a result, no single model to predict severe outcomes of C. difficile infection has arisen as clearly superior or gained widespread acceptance (15). The primary objective of our study was to evaluate the performance of the leading clinical risk prediction models available for C. difficile infection in hospitalized patients.

**TABLE 1:** Existing models for C. difficile infection outcomes and reported area under the receiver operating characteristic curve (AuROC)^*a*^

Study (model name)	C. difficile outcome(s)	Derivation cohort size	Single or multicenter	AuROC
**Belmares et al. (CDD)^*b*^ (** 8 **)**	**Resolution of diarrhea**	**102**	**Single**	**0.89**
Im et al. (10)	Inpatient mortality	396	Single	0.87
Kulaylat et al. (1)	All-cause mortality	2065	Multicenter	0.82
**Lungulescu et al. (CSI)^*b*^ (** 51 **)**	**All-cause mortality, ICU admission, >10-day hospital stay, or colectomy.**	**255**	**Single**	**0.80**
Zilberberg et al. (52)	30-day all-cause mortality	278	Single	0.74
Archbald-Pannone et al. (53)	30-day attributable mortality	362	Single	0.74
**Hensgens et al.^*b*^ (** 16 **)**	**Complicated (all-cause mortality, prolonged ICU stay, or colectomy)**	**395**	**Multicenter**	**0.73**
**Shivashankar et al. (** 17 **)**	**Severe/complicated (hypotension, shock, sepsis)**	**1,146**	**Single**	**0.71**
**Miller et al. (ATLAS)^*b*^ (** 9 **)**	**Response to therapy (“cure”)**	**1,164**	**Multicenter**	**0.71**
Li et al. (54)	Mortality, ICU admission, or colectomy	1,118	Multicenter	0.69
**Na et al. (CDSS)^*b*^ (** 18 **)**	**Contributable mortality or ICU admission, toxic megacolon, or colectomy**	**263**	**Multicenter**	**0.66 (32)**
**Zar et al.^*b*^ (** 15 **)**	**Cure, treatment failure, relapse**	**150**	**Single**	**0.66 (31)**
Kassam et al. (CARDS) (55)	All-cause in-hospital mortality	77,776 (administrative-only database)	Multicenter	0.66
Butt et al. (56)	All-cause mortality	213	Single	0.65
Toro et al. (SSI)^*b*^ (57, 58)	Inpatient mortality and/or ICU admission	51	Single	0.64 (31)
Hu et al.^*b*^ (59)	Recurrence	63	Single	0.62
van der Wilden et al. (RSS) (60)	30-day all-cause mortality, ICU admission, or colectomy	746	Single	0.57 (32)
Drew et al. (RUWA)^*b*^ (61)	Mortality, ICU admission, pancolitis on imaging, or colectomy	81	Single	Not calculated
Neal et al.^*b*^ (62)	Clinical resolution (of symptoms and WBC)	49	Single	Not calculated
Bauer et al. (Hines VA) (63)	Treatment failure	1,105	Multicenter	Not calculated

a ICU, intensive care unit. Models chosen for external validation shown in bold.

b Externally validated (using retrospective data) and/or prospectively validated (in small, single-center studies).

## RESULTS

**FIG 1:**
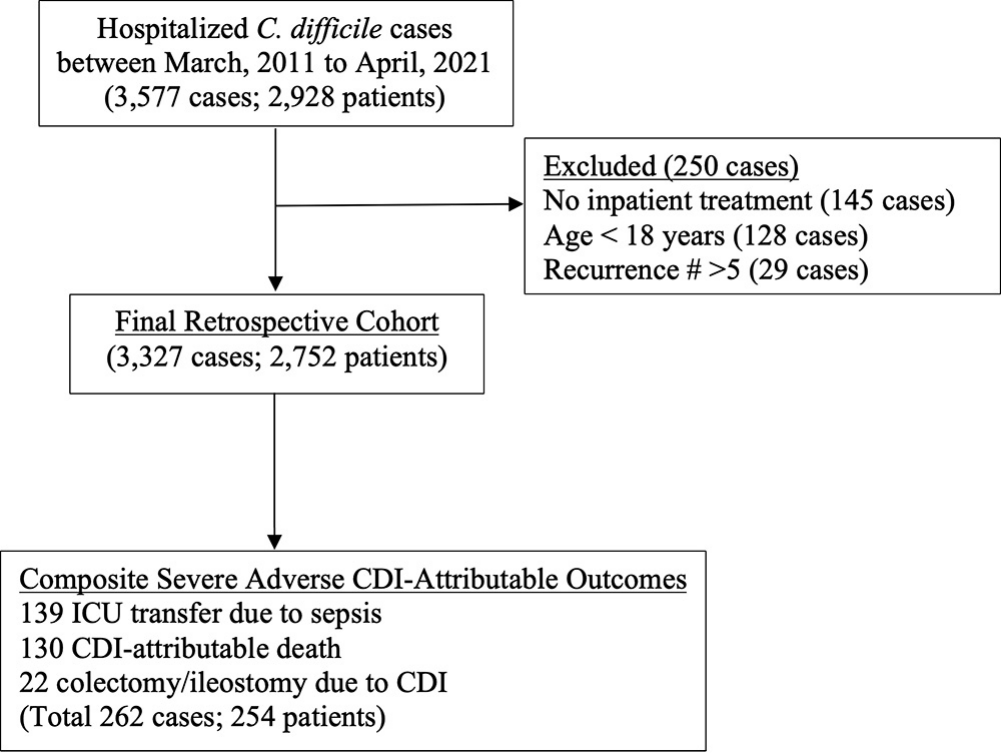
C. difficile infection cohort and composite severe adverse CDI-attributable outcomes. Some patients met >1 exclusion criteria and outcomes were coded as having met ≥1 composite outcome but >1 composite outcomes occurred in some patients.

Three thousand five hundred and seventy-seven hospitalized cases of C. difficile infection were identified between March 2011 and April 2021 that occurred in 2,928 individual patients (Fig. 1). After excluding cases without treatment, age < 18 years, or > 5 recurrent episodes, the final cohort consisted of 3,327 cases in 2,752 individual patients. Baseline characteristics of our cohort were compared to the validation cohorts for the six clinical models (Table 2). Two hundred sixty-two of 3,327 (7.9%) of cases met one or more of the CDI-attributable primary composite outcomes. Clinician reviews indicated that 130/192 (67.7%) of deaths were attributable to CDI and 22/31 (71.0%) of colectomies or ileostomies were due to CDI. 139/295 (47.1%) of transfers to an ICU following CDI diagnosis were due to sepsis according to the validated definition (19). Baseline lab measurements were not available for 356/3,327 (10.7%) creatinine, 378/3,327 (11.4%) white blood cell count (WBC), and 563/3,327 (16.9%) albumin, measurements. Patients with one unavailable measurement at the time of diagnosis were less likely to develop a severe CDI-attributable outcome compared to patients with all measurements (21/609 [3.4%] versus 201/1444 [8.9%]).

**TABLE 2:** Characteristics of UVA Medical Center cohort and derivation cohorts for C. difficile risk scores^*a*^

Characteristic (cohort size)	UVA (*n* = 3,327)	ATLAS (9) (*n* = 1164)^*d*^	Zar (15) (*n* = 150)	Hensgens (16) (*n* = 395)	CDSS (18) (*n* = 263)	CDD (8) (*n* = 102)	Shivashankar (17) (*n* = 1446)
C. difficile cohort setting (yr)	Adult (≥18 yr) inpatients at 645-bed tertiary care hospital, Charlottesville, VA (2011–2021).	Adults (≥16 yr) enrolled in 2 clinical trials: 62 sites in US/Canada (2006–2008) (33), 86 sites in US/Canada/Europe (2007–2009) (34), total ~36% outpatients.	Adult (≥18 yr) inpatients at 200-bed acute care hospital in Chicago, IL (1994–2002).	Prospective inpatients of any age admitted to 9 centers in The Netherlands (2006–09).	Adult (≥18 yr) prospective inpatients at 1 tertiary care cohort in Boston, MA (2004–06).	Inpatients of any age admitted to a 472-bed tertiary care Hines Veterans Affairs hospital in Chicago, IL (2003–04).	1 center retrospective cohort in Rochester MN (2007–2010).
Model development; validation	External Validation Cohort	Multivariable Logistic Regression derived on first trial cohort that was validated on the second. Final validation on the pooled cohort (*n* = 1,105).	Ad hoc score developed to stratify disease severity for randomized clinical trial.	Multivariable Logistic Regression; bootstrapping (*n* = 200) and shrinkage factor. External validation at 1 center (2009–2011).	Multivariable Logistic Regression; Validated using cohorts in Dublin, Ireland (2007–09) and Houston, TX (2006–2010).	Empiric score developed based on variables described in the literature, validated using cohort.	Multivariable logistic regression; internal validation.
Age (mean)	63.0	62.5	58.5	65.0 (median)	66.5	68.25	62.5 (median)
Male sex	1,686 (50.7)	462/1105 (41.8)	82 (54.6)	220 (56)	131 (49.8)	NA	220 (56)
Charlson comorbidity index	1.78 (mean)	NA	NA		3.28 (mean)	1.68 (mean)	NA
0	1,121 (33.7)			59 (14.9)			
1–2	963 (28.9)			150 (40.0)			
3–4	659 (19.8)			120 (30.4)			
≥5	584 (17.6)			64 (16.2)			
Antibiotic exposure^*b*^ (<2 mo)	2830 (85.1)	NA	150 (100)	336 (85.0)	NA	NA	614 (42.5) before or after CDI
Fluoroquinolones	624 (18.8)						
Cephalosporins	1021 (30.7)						
Carboxy/ureidopenicillins	921 (27.7)						
Macrolides/clindamycin	315 (9.5)						
Antistaphylococcal/aminopenicillins	614 (18.4)						
Acid suppression (<2 mo)	2,246 (67.5)	NA	NA		198 (75.2)	NA	NA
PPI	1,579 (47.5)			251 (63.5)			
H2-RA	1212 (36.4)						
PPI + H2-RA	545 (16.4)						
Immunosuppression (<6 mo)	721 (21.7)	NA	NA	172 (43.5)	117 (44.5)	NA	NA
CDI diagnosis method		All patients had + Toxin EIA or + Cytotoxicity Assay		All patients had + Toxin EIA or + Cytotoxicity Assay			
PCR	3251 (97.7)		0 (0)		0 (0)	0 (0)	1446 (100)
Toxin EIA	76 (2.3)		150 (100)		263 (100)	102 (100)	0 (0)
Cytotoxicity assay	0 (0)		0 (0)		0 (0)	0 (0)	0 (0)
NHSN classification		746 (64.0) inpatient; 418 (35.9) outpatient	NA		NA		NA
Hospital onset	1,745 (52.4)			283 (71.6)		25 (24.5) community-onset	
Community onset-HCFA	526 (15.8)						
Community-acquired	1,056 (31.7)						
Primary CDI episode	2,740 (82.4)	978 (84.0)	NA	NA	221 (84.0)	99 (97.1)	NA
Ribotype 027 (n/available)	10/97 (10.3)^*e*^	292/814 (35.9)	NA	17/207 (8.2)		60% of isolates	NA
Treatment							NA
Metronidazole (PO or IV)	1,147 (34.5)	0 (0.0)	79 (52.7)	293 (74.2)	247 (93.9)	93 (91.2)	
Vancomycin	1,023 (30.7)	592 (50.9)	71 (47.3)	10 (2.5)		9 (8.8)	
Vancomycin + Metronidazole	1,150 (34.6)	0 (0.0)	0 (0)	47 (11.9)		0 (0)	
Fidaxomicin	7 (0.2)	572 (49.1)	0 (0)	0 (0)		0 (0)	
No Treatment	0 (0.0)	0 (0.0)	0 (0)	47 (11.9)		0 (0)	
CDI outcomes							
ICU admission	295 (8.9)	NA	NA	NA	NA	NA	386 (2.7)
ICU admission for sepsis^*c*^ and/or CDI complications	139 (4.2)	NA	NA	3 (0.8)	32 (12.2)	NA	NA
Colectomy/ileostomy	22 (0.7)	NA	0 (0)	5 (1.3)	9 (3.4)		31 (2.7)
30-day all-cause mortality	249 (7.5)	37/623 (5.9)	8 (5.3)	65 (16.5)	NA	6 (5.9)	102 (8.9)
90-day all-cause mortality	389 (11.7%)						
CDI attributed mortality	130 (3.9)	NA	NA	38 (9.9)	11 (4.2)	2 (2.0)	NA
Complicated CDI outcome (as defined by study)	262 (7.9)	77 (6.6)	14 (9.3)	46 (11.9)	NA	9 (8.8)	487 (33.7)

a Data presented as *n*/total (%) or *n*/available (%). SD, standard deviation; NA, not available; approximately, approximate; mo, months; PPIs, proton pump inhibitors; H2-RA, Histamine type-2 receptor antagonists; EIA, enzyme immunoassay; GDH, glutamate dehydrogenase; HCFA, health care facility-associated; PO, per os; IV, intravenous.

b Each patient could have received more than one class of antimicrobials within 2 months of enrollment.

c In the UVA cohort, defined as ICU transfer due to sepsis (based on validated definition by Rhee et al. (23)), CDI-attributable in-hospital mortality, or in-hospital CDI-attributable colectomy/hemi-colectomy/diverting ileostomy following C. difficile diagnosis.

d For ATLAS, data for the 967/1164 patients which lacked missing variables and were used for model development were not reported; characteristics of the full cohort is shown.

e Data from an unpublished convenience sampling of 97 C. difficile isolates from clinical stool specimens at UVA from August 2018 to April 2019 (personal communication, C.A.W.).

**FIG 2:**
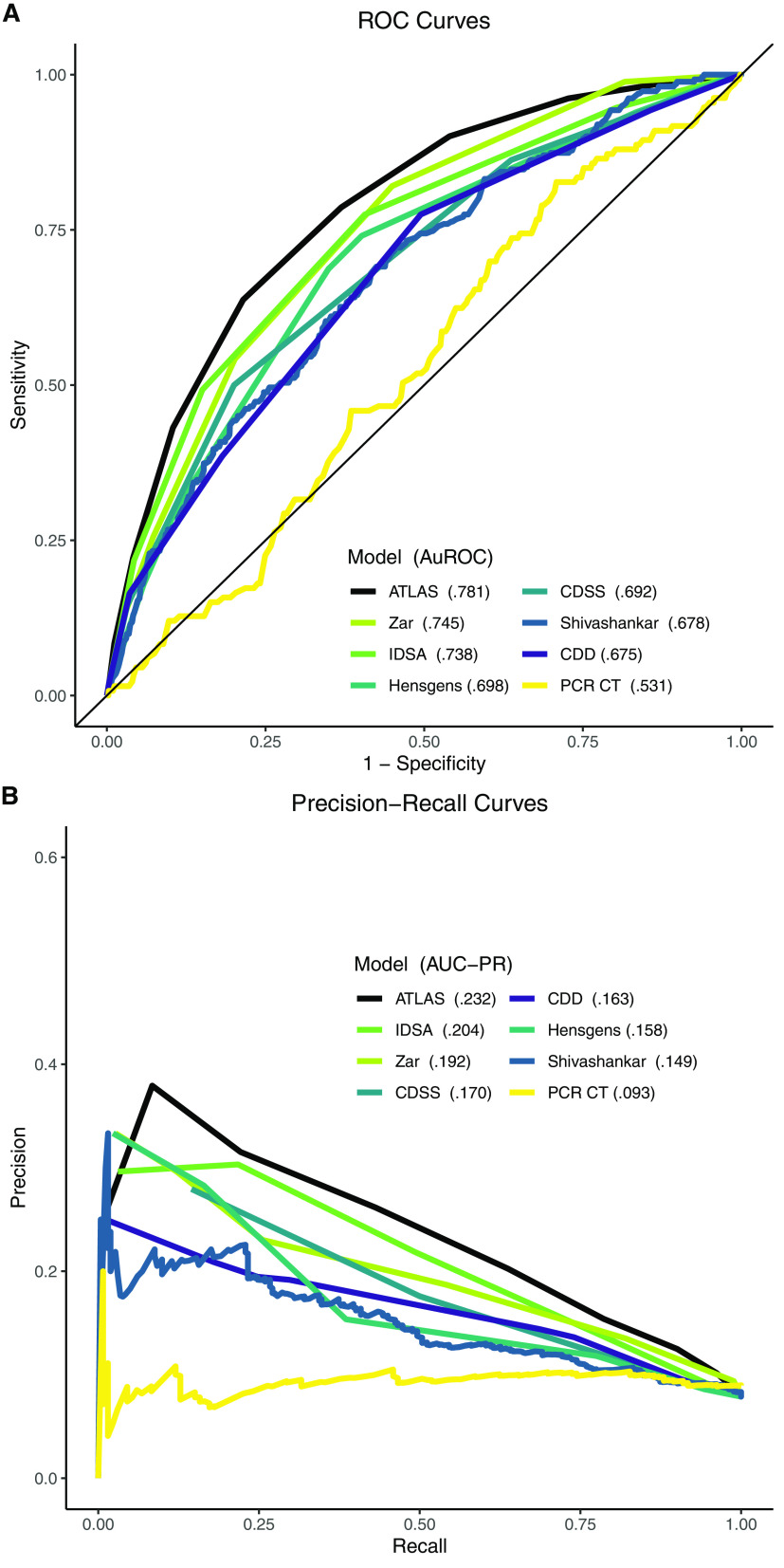
Receiver operating characteristic (A) and precision-recall curves (B) with area under the curves (AuROC and AUC-PR, respectively) for C. difficile risk models. PCR cycle threshold (CT) data only available for 1,484/3,327 cases.

**FIG 3:**
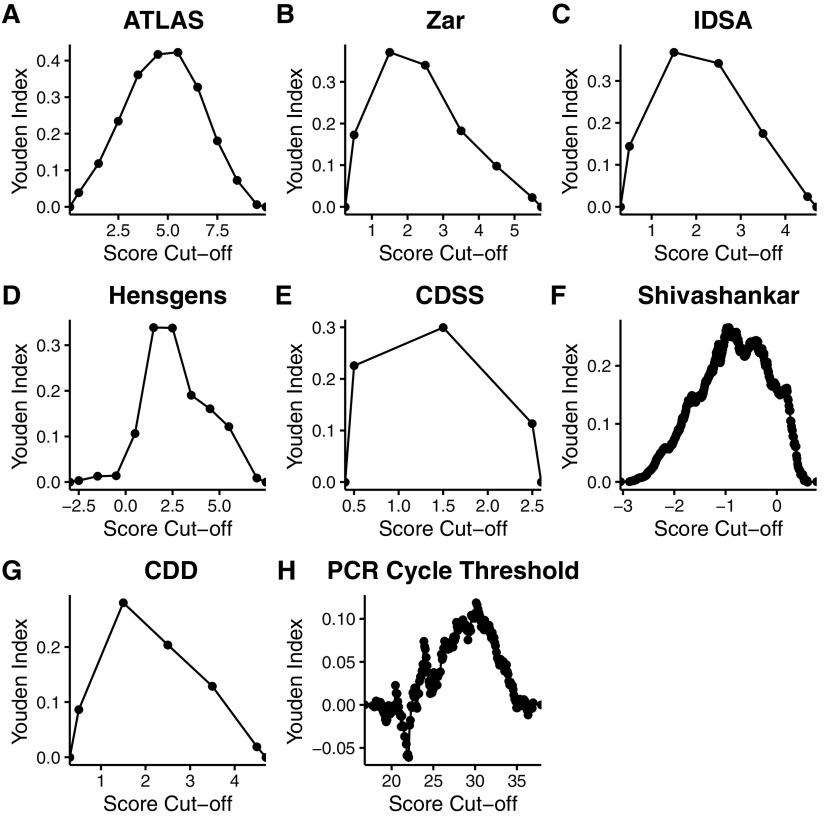
Youden indices for C. difficile severity score cutoffs. Youden Index is equal to 0 for tests with poor diagnostic accuracy, equal to 1 for a perfect test, and assigns equal weight to sensitivity and specificity.

Median (interquartile range) scores among the 262 cases with severe attributable outcomes of CDI versus the remaining 3,065 cases for each of the models were: ATLAS (outcomes: 6 [5–7] versus not: 4 [2–5]), IDSA (2 [2–3] versus 1 [1–2]), Hensgens (3 [1–4] versus 1 [1–3]), CDSS (1.5 [1v2] versus 1 [0v1]), Shivashankar (−0.555 [−1.038 to −0.010] versus −1.020 [−1.54 to −1.02]), and CDD (2 [2–3] versus 1 [1–2]). The ATLAS Score achieved the highest AuROC of 0.781 (Fig. 2) with a maximum F1 of 0.326 at a cutoff ≥7 (Table 3). The maximal Youden Index for ATLAS (0.422) occurred at a cutoff ≥6 (Fig. 3), corresponding with a true positive rate (sensitivity) of 63.7% and a false positive (type 1 error) rate of 21.5%. ATLAS performed significantly better than the next highest AuROC (Zar 0.745; 95% confidence interval for the AuROC difference 0.0094–0.0622: *P = *0.008). Next, in descending order of AuROC were the IDSA Severity Criteria (0.738), Hensgens score (0.698), CDSS (0.692), Shivashankar (0.678), and CDD (0.675). PCR cycle threshold performed the worst, with an AuROC slightly above 0.5 (0.531).

**TABLE 3:** Performance comparison of scoring tools^*a*^

Risk score	Cut-off (*n* patients, %)^*b*^	# Successes, failures	Sens. (%)	Spec. (%)	Positive predictive value (%)	Negative predictive value (%)	F1	AuROC (95% CI)	*P* (vs. ATLAS)^*b*^	AUC-PR
ATLAS	0 (130, 3.9)							0.781 (0.756–0.807)		0.232
	1 (283, 8.5)	390, 2,937	99.6	4.21	8.16	99.2	0.151			
	2 (433, 13.0)	667, 2,660	98.5	13.3	8.85	99.0	0.162			
	3 (590, 17.7)	1,088, 2,239	96.2	27.3	10.2	98.8	0.184			
	4 (553, 16.6)	1,646, 1,681	90.1	46.0	12.5	98.1	0.219			
	5 (513, 15.4)	2,139, 1,188	78.6	63.1	15.4	97.2	0.258			
	**6 (393, 11.8)**	2,574, 753	63.7	78.5	20.2	96.2	0.307			
	7 (248, 7.5)	2,859, 468	43.1	89.6	26.2	94.9	0.326			
	8 (126, 3.8)	2,997, 330	22.1	95.9	31.5	93.5	0.260			
	9 (50, 1.5)	3,051, 276	8.40	98.8	37.9	92.7	0.138			
	10 (8, 0.24)	3,061, 266	0.76	99.8	25.0	92.2	0.015			
Zar	0 (567, 17.0)							0.745 (0.723–0.768)	**0.008**	0.192
	1 (1167, 35.1)	823, 2,504	98.9	18.4	9.45	99.5	0.171			
	**2 (833, 25.0)**	1,902, 1,425	82.1	55.0	13.5	97.3	0.231			
	3 (469, 14.1)	2,589, 738	54.2	79.8	18.7	95.3	0.278			
	4 (183, 5.5)	2,908, 419	25.6	92.7	23.0	93.6	0.242			
	5 (87, 2.6)	3,021, 306	12.2	97.5	29.6	92.9	0.173			
	6 (21, 0.63)	3,058, 269	2.67	99.5	33.3	92.3	0.049			
IDSA/SHEA	0 (620, 18.6)							0.738 (0.12–0.765)	**0.005**	0.204
	1 (1258, 37.8)	854, 2,473	94.7	19.8	9.16	97.7	0.167			
	**2 (859, 25.8)**	2,022, 1,305	77.5	59.3	14.0	96.9	0.237			
	3 (402, 12.1)	2,733, 594	49.2	85.0	21.9	95.1	0.303			
	4 (161, 4.84)	2,991, 336	21.8	95.7	30.3	93.5	0.253			
	5 (27, 0.81)	3,054, 273	3.05	99.4	29.6	92.3	0.055			
Hensgens	−1	311, 3,016	99.6	1.63	7.97	98.0	0.148	0.738 (0.712–0.765)	**<0.0001**	0.158
	1	759, 2,568	93.9	16.7	8.79	97.0	0.161			
	2	2,028, 1,299	74.0	59.8	13.6	96.4	0.230			
	4	2,814, 513	29.8	89.3	19.2	93.7	0.233			
CDSS	0 (1148, 34.5)							0.692 (0.666–0.719)	**<0.0001**	0.170
	1 (1434, 43.1)	1,338, 1,989	86.3	36.3	10.4	96.9	0.185			
	**2 (609, 18.3)**	2,582, 745	50.0	80.0	17.6	94.9	0.260			
	3 (136, 4.1)	3,005, 322	14.5	96.8	27.9	93.0	0.191			
Shivashankar	−2.0	519, 2,808	98.9	8.32	8.45	98.8	0.156	0.678 (0.645–0.711)	**<0.0001**	0.149
	−1.4	1,124, 2,203	87.4	29.2	9.54	96.4	0.172			
	**−0.930**	1,969, 1,358	69.1	57.5	12.2	95.6	0.207			
	−0.4	2,058, 1,269	44.7	79.3	15.6	94.4	0.233			
CDD	0 (454, 13.6)							0.675 (0.646–0.704)	**<0.0001**	0.163
	1 (1154, 34.7)	686, 2,641	94.3	14.3	8.6	96.7	0.158			
	**2 (1061, 31.9)**	1,752, 1,575	77.5	50.5	11.8	96.3	0.205			
	3 (506, 15.2)	2,609, 718	38.5	81.8	15.3	94.0	0.220			
	4 (134, 4.0)	2,999, 328	16.4	96.4	28.3	93.1	0.208			
	5 (18, 0.54)	3,059, 268	2.3	99.6	33.3	92.3	0.043			
PCR cycle threshold	32.0	402, 1,082	87.2	21.2	9.82	94.4	0.177	0.531 (0.483–0.579)	**<0.0001**	0.093
	**30.0**	506, 978	81.2	29.5	10.2	94.1	0.181			
	28.0	614, 870	70.7	38.5	10.2	93.0	0.178			
	26.0	708, 776	58.6	46.6	9.76	92.0	0.167			

a Sens., sensitivity; Spec., specificity; AuROC, area under the receiver operating characteristic curve; AUC-PR, area under the precision-recall curve; CI, confidence interval.

b Calculations based on ≥ cutoff, except for PCR cycle threshold (≤ cutoff). Cutoffs that represent the maximum Youden Index are shown in bold.

**FIG 4:**
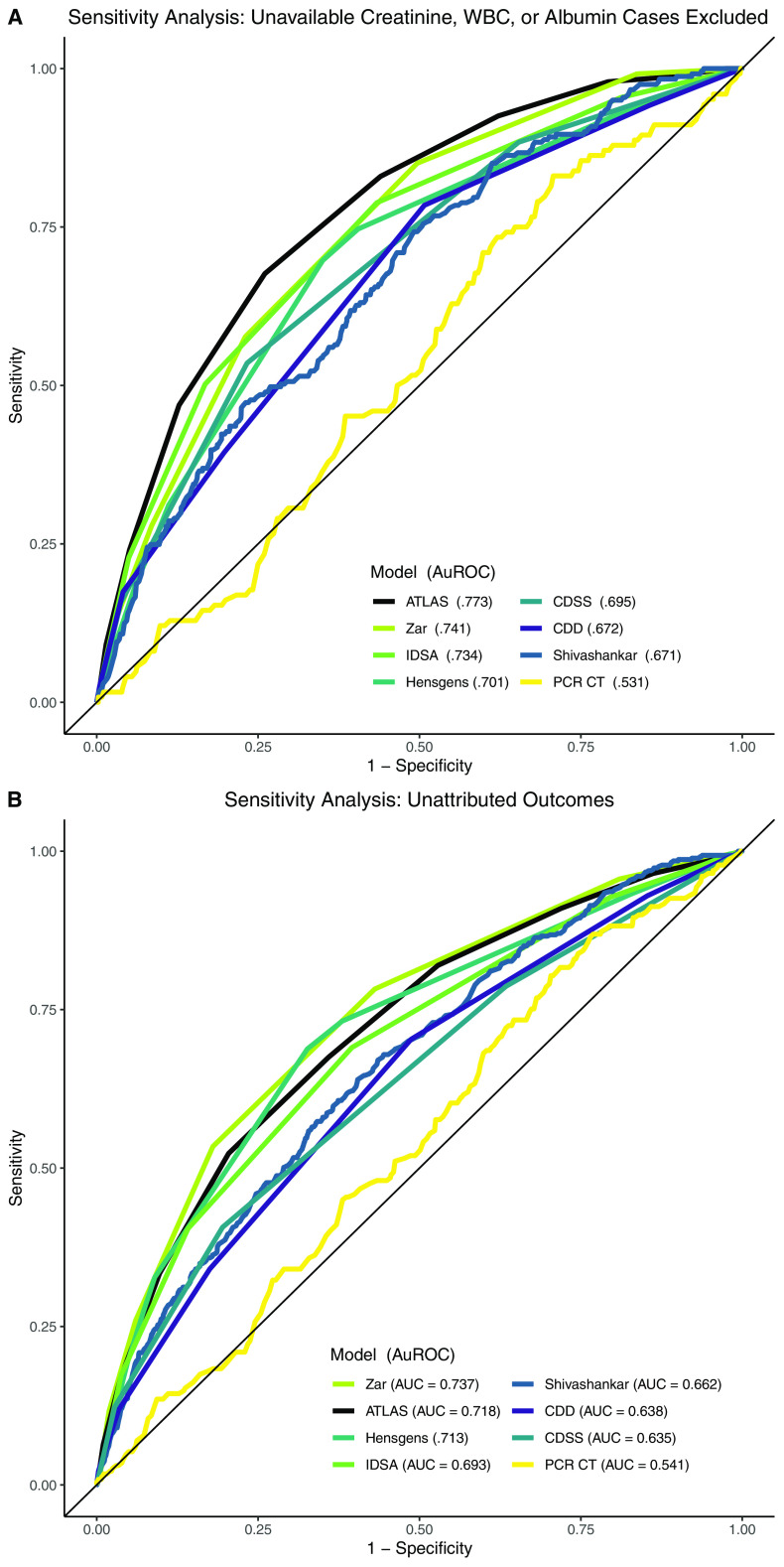
Receiver operator curves with area under the receiver operating characteristic curve (AuROC) for C. difficile risk models using the full cohort (A) minus 609 patients with unavailable white blood cell count, creatinine, or albumin measurements and (B) an unattributed composite outcome (all-cause inpatient mortality, all-cause colectomy/ileostomy, and/or all-cause ICU transfer). PCR cycle threshold (CT) data only available for 1,484/3,327 cases.

Two sensitivity analyses were performed: (i) excluding 609 cases without available creatinine, WBC, or albumin measurements at the time of diagnosis (Fig. 4A) and (ii) using a composite outcome without clinical attributions (all-cause inpatient mortality, all-cause ICU transfer, or all-cause colectomy/ileostomy) (Fig. 4B). Both demonstrated similar trends with the exception that Zar, not ATLAS, was the leading model for predicting unattributed outcomes (Zar AUC 0.737; ATLAS 0.718; Hensgens 0.713; IDSA/SHEA 0.693; Shivashankar 0.662; CDD 0.638; CDSS 0.635; PCR cycle threshold 0.541).

## DISCUSSION

These data support the notion that C. difficile clinical risk scores have a performance ceiling, especially early in the disease course. While ATLAS, Zar, and the IDSA Severity criteria all fell within an AuROC range ≥ 0.7-0.8 that could be considered clinically useful (20), AUC may be misleading in settings such as CDI where outcomes are unbalanced. For example, ATLAS achieved the highest maximal Youden index of 0.422 at a score cutoff of ≥6, however, the positive predictive value (20.2%) at this cutoff was strikingly low. Therefore, the clinical utility of ATLAS to predict attributable outcomes of infection remains unclear.

There are important limitations to this analysis. First, while this is one of the largest single-center cohorts used for C. difficile model validation, it does not necessarily indicate generalizability to other tertiary care settings. ATLAS did not show stable performance across one multi-institution cohort (13) and should be validated in other settings such as community hospitals or outpatient prior to widespread adoption. Second, C. difficile infection occurs inherently in patients that are medically complex (i.e., on antibiotics for other causes, immunosuppressed, multiple comorbidities) and clinician determination of attributable causes for C. difficile complications is often difficult, subjective, and lacks inter-rater reliability (21, 22). ICU transfers meeting a validated electronic definition for sepsis was therefore chosen to minimize the time and subjectivity of clinician reviews. However, this method has not been previously studied in the context of CDI and requires further validation. Third, there were a significant proportion of unavailable measurements that we chose to score with zero points as a practical and inclusive approach to test the “real world” prognostic value of each model. Interestingly, model performance appeared to improve through this imputation method versus omitting cases. Since patients with at least one unavailable measurement had nearly a third the rate of severe outcomes; we hypothesize that the decision to not check a full completement of labs at diagnosis implied an overall favorable prognosis but may have underutilized important prognostic information in some cases. Fourth, electronic-only data gathering did not allow specific components of the CDD (imaging findings) and Hensgens (admission reason) models to be included; however, this did not appear to significantly impact performance compared with other retrospective validation studies (i.e., CDD AUC at UVA: 0.675 versus AUC reported by Perry et al.: 0.620 [13]; Hensgens AUC at UVA: 0.698 versus AUC reported by Beauregard-Paultre and van Beurden et al.: 0.630–0.680 [11, 14]).

Two recent studies attempting to validate these models using smaller, single-center cohorts failed to identify a significantly superior model (11, 13); however, our data showed that ATLAS performed significantly better than other major models. ATLAS was originally derived using two pooled clinical trial cohorts for comparing fidaxomicin and vancomycin treatment (23, 24) that excluded patients with “life-threatening”/fulminant infection or toxic megacolon and approximately a third of the ATLAS cohort were outpatients (25). Despite these limitations, ATLAS appears externally valid to our relatively sicker, inpatient CDI cohort. Similarly, ATLAS performed well at hospitals in Mexico where severe outcomes were more prevalent (i.e., 17.6% 30-day all-cause mortality, 3.9% colectomies) (26). Our ATLAS AuROC was higher than that originally reported by Miller et al. (0.78 versus 0.71), which may be explained by differences in the outcome definition (ATLAS constrained to “clinical failure” defined as a lack of marked reduction of diarrhea and/or need for additional C. difficile therapy based on investigator opinion) (9).

There are several reasons why ATLAS may be superior to other models in our study. ATLAS was derived from one of the largest cohorts and combines 5 robust factors, each of which have repeatedly shown to be important predictors of severe infection in other studies (27). In addition, the ATLAS criteria can be extracted from the electronic medical record with relative ease, objectivity, and fidelity, which was not necessarily the case with the other models, which relied on imperfect billing/coding data (e.g., presence of ileus or megacolon in CDD, IDSA/SHEA) or factors that could not be extracted electronically (CDD, Hensgens).

ATLAS performed significantly worse (AuROC 0.781 versus 0.718) in the *post hoc* analysis to predict severe outcomes not attributable to CDI. While ATLAS and Zar criteria are very similar, ATLAS has an important feature which Zar lacks: non-CDI antibiotics during CDI treatment. Cases with non-CDI antibiotics during CDI treatment were about twice as likely to develop the composite attributable outcome (189/1888, 10%) compared to cases without additional antibiotics (73/1439; 5.1%). Since concomitant antibiotics is a well-established independent risk factor that is specific to CDI (28, 29), this factor may be particularly important for predicting outcomes attributable to CDI.

While the Youden Index is often used to select an “optimal” cutoff point for diagnostic markers when equal weight is given to sensitivity and specificity, choosing a clinically relevant ATLAS score must take into account its intended use, outcome prevalence (and effects on positive/negative predictive value), and tradeoffs of minimizing false positives or false negatives. For example, if the goal is to correctly identify CDI patients very likely to develop severe complications at the expense of sensitivity/recall (e.g., decisionmaking for an irreversible, morbid intervention such as total colectomy), an ATLAS cutoff ≥9 might be reasonable, which corresponds with a relatively low false positive rate 1.2%. On the other hand, in situations where negative predictive value is of highest importance (e.g., identifying CDI inpatients for early discharge), an ATLAS cutoff of <4 would afford >98% negative predictive value (assuming outcome prevalence 7.9%).

The IDSA/SHEA Guideline definition for severe infection using WBC and creatinine alone (30) has historically been a poor predictor of outcomes (17). The addition of up to 3 empirical “points” for each of hypotension, shock, and ileus/megacolon (≥1 criterion previously termed “complicated” (30) or “fulminant” infection (15, 16)) did have a maximal positive predictive value (30.3% with cutoff ≥4) comparable to ATLAS (37.9% with cutoff ≥9), however, our analysis nonetheless indicates there is potential room for improvement in the Guideline-recommended Severity Classifications.

PCR-based C. difficile testing, now used to diagnose >80% of US cases (31), is highly sensitive but cannot differentiate colonization from infection. The PCR cycle threshold has an inverse correlation with C. difficile organism burden. A low cycle threshold (i.e., high organism burden) correlates with toxin EIA positivity (6), and disease severity at some centers (32, 33) but not all centers (34). Our data showed that PCR cycle threshold was poorly predictive for severe outcomes, which is in keeping with recent work demonstrating that the immune response, not bacterial burden, mediates severity (35). Variable correlation between cycle threshold and severe disease could be explained by the significant variations in C. difficile strain virulence observed at different studies (e.g., binary toxin gene prevalence ranges 0.2% to 48% (36)) and/or variations in quantitative toxin levels (not typically measured).

To augment future iterations of ATLAS or other clinical-only models, evaluating novel biomarkers, either host or pathogen factors, would be a logical next step. For example, the CDI-specific host immune response (37) is recognized to play a central role in pathogenesis (38) and data suggest specific biomarkers could be valuable in conjunction with clinical markers (e.g., with an AUC up to 0.91 in one study) (35). Additionally, C. difficile ribotype 027 and other binary toxin-producing strains independently predict disease severity (39–41) and may be useful adjuncts to clinical risk assessment. Score calculations generated entirely from the electronic medical record using data available within 48 h of diagnosis, and the use of a unique validated electronic definition for sepsis also have important implications on the feasibility of future automated research applications and clinical decision support.

## MATERIALS AND METHODS

### Study population.

A retrospective cohort of hospitalized adult patients with C. difficile infection was developed at University of Virginia Medical Center, a 645-bed, tertiary care academic hospital. Hospitalized cases were identified based on at least 1 positive C. difficile PCR (PCR; GeneXpert; Cepheid, Sunnyvale, CA) test between March 2011 and April 2021. CDI cases in children were rare (<5%) and were excluded due to differences in testing recommendations and given that measures of clinical status (e.g., Charlson) and the validated sepsis definition were not applicable in patients <18 years. Also excluded were cases with > 5 prior recurrent episodes, and those that did not receive active treatment (oral vancomycin, IV or oral metronidazole) while inpatient. This study received approval from the University of Virginia Institutional Review Board (#20082).

### Data collection/risk score calculation.

Twenty models for C. difficile infection severity were reviewed from the literature (Table 1) and six (Age Treatment with Systemic Antibiotics Leukocyte Count Albumin and Serum Creatinine [ATLAS] (9), C. difficile Disease [CDD] [8], Zar et al. [42], Hensgens et al. [43], Shivashankar et al. [44], C. difficile Severity Score (CDSS) [45]; Table 4) were chosen for validation based on their performance, prominence in the literature, derivation cohort size, prior validation, parameters that could be reliably gathered from the electronic medical record at the time of diagnosis, and ≥4 ordinal scores that could be fitted to an ROC curve. For IDSA Severity, one point was empirically assigned for each criterion for Severe and Fulminant infection from the Updated 2017 IDSA/SHEA Guidelines (15, 16). In addition, we were interested if real-time C. difficile PCR cycle threshold data (as an inverse measure of fecal organism burden) could be independently useful for predicting severe outcomes since high burden/low cycle threshold (i.e., ≤28.0) has been shown to correlate with worse outcomes associated with CDI (6, 32, 46).

**TABLE 4:** Six major clinical scoring methods to predict severe cdi-attributable outcomes^*a*^

Model	Clinical criteria (points)	Outcome(s)	Score range
ATLAS (9)	Age, non-CDI systemic antibiotics, creatinine, WBC, albumin (0–2 points for each)	“Clinical failure” (lack of marked diarrhea reduction or need for further C. difficile therapy)	0–10
CDD (8)	Fever (1), ileus (1), SBP < 100 (1), WBC (2), abdominal imaging findings (2; excluded).	Diarrhea resolutio*n* ≤6 days after therapy initiation	0–5
Zar (15)	Age (1), albumin (1), ICU (2), temp (1), pseudomembranes (2; excluded), WBC (1)	Cure, treatment failure, relapse	0–6
IDSA/SHEA (11)	WBC (1), creatinine (1), hypotension (1), shock (1), ileus/megacolon (1)	Based on “non-severe” vs. “severe” (WBC > 1500 or Cr ≥ 1.5) and “fulminant” IDSA/SHEA definitions (11, 12)	0–5
Shivashankar (17)	Age (x0.01), WBC (0.81), narcotic use (0.77), H2-antogonist or PPI (0.63), creatinine > 1.5x baseline (0.52)	Severe/complicated (hypotension, shock, sepsis)	−2.88–0.61
CDSS (18)	Age, creatinine, WBC (1 point each)	Contributable mortality, ICU admission, or attributable toxic megacolon/colectomy	0–3
Hensgens (16)	Age (3), diagnosis in ICU (3), recent abdominal surgery (−3), hypotension (2), admission for diarrhea (2; excluded)	Prolonged ICU admission, attributable colectomy or 30d mortality	−3–8

a ICU, intensive care unit.

Baseline clinical and outcome data were gathered electronically from the University of Virginia Clinical Data Warehouse, a database containing billing/coding, clinical, pharmacy, and laboratory data from the Epic electronic medical record. Baseline clinical data included the closest available measurement within ±48 h of the index positive C. difficile PCR specimen collection time. In patients with multiple repeat positives during a hospitalization, time of diagnosis was based upon the initial positive result. If multiple laboratory measurements were available, the maximum white blood cell count (WBC), creatinine, and minimum albumin measurements were used. Unavailable measurements were assigned 0 points (i.e., 0 points assigned for ATLAS albumin criterion if not performed at diagnosis). The Charlson Comorbidity Index and the presence of ileus or megacolon were collected using International Classification of Diseases (ICD) coding data (47). National Healthcare Safety Network surveillance definitions were assigned to each case: hospital-onset CDI (HO-CDI), hospital-onset health care-facility-associated (HO-HCFA), or community onset (CO-CDI) (48). Shock was defined by the need for vasopressors. Immunosuppressive medications were defined as ≥60mg oral daily prednisone or equivalent systemic corticosteroid, azathioprine, rapamycin derivatives, cyclosporine, tacrolimus, or mycophenolate. Antimotility medications were defined as loperamide, diphenoxylate, oral opium, or bismuth subsalicylate.

Risk model scores were calculated based on parameters gathered electronically for each case at the time of diagnosis. The following features could not be reliably gathered from the electronic record and so were omitted from score calculations: specific computed tomography abdominal imaging findings (thickened colonic wall, dilation, or ascites) from the CDD score (8), diarrhea as the reason for admission from the Hensgens score (43), and presence of pseduomembranes on endoscopy from the Zar criteria (42). Cycle threshold values from the GeneXpert (Cepheid, Sunnyvale CA) PCR platform were available from archived data for 1,484 cases that occurred between November 2013 and June 2018. Beginning February 2020, UVA Health transitioned from PCR-only testing to multistep PCR with reflex (if PCR+) to toxin enzyme immunoassay (Alere C. DIFF QUIK CHEK COMPLETE), with both results submitted to the treating clinician.

### Outcomes.

Severe adverse outcomes attributable to CDI were defined as ICU transfer due to sepsis, CDI-attributable mortality, or colectomy, hemi-colectomy, or diverting ileostomy due to CDI. Mortality and surgery attributions were determined by an Infectious Diseases specialist with expertise in C. difficile (G.R.M.). ICU transfers due to sepsis were categorized electronically using a validated definition by Rhee et al. (19), based on the Sepsis-3 criteria (49) (evidence of presumed serious infection + acute organ dysfunction). Non-attributable outcomes were also collected including all-cause 30-day and 90-day mortality.

### Data analysis.

Using the risk scores calculated based on patient demographics and clinical characteristics, we classified cases into score-specific strata and calculated the standard diagnostic test summary indices (sensitivity, specificity, positive predictive value, and negative predictive value) for each stratum. The area under the receiver operating characteristic curve (AuROC) and area under the precision-recall curve (AUC-PR) for each model were then calculated from these score-specific diagnostic test summary indices. The Youden Index (sensitivity + specificity − 1) was calculated as an overall measure of diagnostic effectiveness and as one method to identify an optimal cutoff that balances sensitivity and specificity (Youden ranges 0–1, with 0 indicating a useless test and 1 indicating no false positives or false negatives) (50).

Delong’s test of variance was used to calculate two-sided statistical comparisons of the highest performing model AuROC against each of the others. F1 scores (harmonic mean of precision (positive predictive value) and recall (sensitivity)) were calculated for each model. Analyses were performed using statistical software R, version 4.1.2 (R Core Team, Vienna, Austria) and the following R packages: dpylr (51), comorbidity (52), ROCit (53), pROC (54), and PRROC (55).
